# Clinical and Patient-Reported Outcomes Following Total Knee Revision for Arthrofibrosis Using Modern Rotating Hinged Knee Arthroplasty and Preoperative Radiation Therapy: A Single-Institution Retrospective Analysis

**DOI:** 10.1016/j.adro.2026.102110

**Published:** 2026-07-31

**Authors:** Jason Liu, Cody C. Green, George J. Haidukewych, Amish P. Shah, Patrick Kelly, Justin Rineer

**Affiliations:** a College of Medicine, University of Central Florida, Orlando, Florida; b Orlando Health Jewett Orthopedic Institute, Orlando, Florida; c Department of Radiation Oncology, Orlando Health Cancer Institute, Orlando, Florida

## Abstract

**Purpose:**

Arthrofibrosis is a debilitating complication following total knee arthroplasty (TKA) characterized by excessive fibroproliferation leading to restricted range of motion (ROM) and impaired function. Radiation therapy (RT) has demonstrated efficacy in modulating fibroblast activity in benign conditions, yet patient-reported outcomes following RT-assisted management of arthrofibrosis remain poorly defined. This study evaluates clinical and patient-reported outcomes in patients undergoing revision TKA for severe arthrofibrosis augmented with preoperative RT.

**Methods and Materials:**

In this retrospective study, patients undergoing revision TKA for severe arthrofibrosis with adjunctive preoperative RT between 2023 and 2025 were identified. All patients received a single fraction of 8.0 Gy external beam RT within 24 hours prior to surgery. Clinical outcomes included passive ROM measured preoperatively, at 6 weeks, and at final follow-up. Patient-reported outcomes were assessed using Patient-Reported Outcomes Measurement Information System (PROMIS) Physical Function, PROMIS Pain Interference, PROMIS Global Physical Health, and Knee injury and Osteoarthritis Outcome Score for Joint Replacement instruments. Paired *t* tests compared pre- and postoperative outcomes.

**Results:**

Thirteen patients were included with a mean follow-up of 19 weeks. Mean total arc of motion improved from 69.5° preoperatively to 94.5° at 6 weeks and 98.3° at final follow-up (*P* < .01). PROMIS Physical Function improved by a mean of 7.8 points (*P* = .013), PROMIS Pain Interference decreased by 10.1 points (*P* = .017), and Knee injury and Osteoarthritis Outcome Score for Joint Replacement improved by 25.3 points (*P* = .004). Patient-reported outcome data were available for 12 patients. PROMIS Global Physical Health demonstrated a nonsignificant improvement. No radiation-related adverse events or reoperations were observed.

**Conclusions:**

Preoperative RT appears to be a safe and effective adjunct in the management of severe arthrofibrosis following TKA, resulting in meaningful improvements in ROM and patient-reported functional outcomes. These findings support an expanding role for RT in benign musculoskeletal fibroproliferative disorders.

## Introduction

Arthrofibrosis is a disabling complication of total knee arthroplasty (TKA), occurring in 3% to 10% of patients, and is characterized by excessive periarticular scar formation that restricts range of motion (ROM) and leads to persistent pain and functional limitations.^1^, ^2^, ^3^ Traditional management strategies, including physical therapy, manipulation under anesthesia, arthroscopic lysis of adhesions, and revision surgery, can improve motion, yet recurrence and subsequent interventions are frequent, with reported rates highest following revision TKA.^4^ These limitations have prompted investigation into adjunctive biologic strategies to modulate the fibroproliferative response.

Radiation therapy (RT) has been successfully employed in other fibroproliferative disorders such as heterotopic ossification and keloid scars, where it suppresses fibroblast proliferation and reduces recurrence rates.^5^^,^^6^ Mechanistic studies demonstrate that radiation suppresses fibroblast proliferation, contractility, and extracellular matrix deposition.^7^ Animal models of posttraumatic joint contracture further suggest that proliferating myofibroblasts in the early perioperative period are particularly radiosensitive.^8^ Preoperative RT was selected over postoperative protocols to target these proliferating myofibroblasts at the earliest possible stage of the wound-healing cascade, theoretically inhibiting the fibrotic response before significant extracellular matrix deposition occurs in the immediate postoperative period. These findings provide a biologic rationale for the use of preoperative RT to mitigate arthrofibrosis.

Clinical experience with RT for arthrofibrosis following TKA has been limited to small case series and retrospective cohorts, which have reported encouraging improvements in ROM.^9^, ^10^, ^11^, ^12^ However, these studies have focused primarily on objective motion outcomes, with limited evaluation of patient-reported functional recovery.

This retrospective study evaluates both objective ROM and validated patient-reported measures in patients undergoing revision TKA for severe arthrofibrosis with adjunctive preoperative RT, framing RT as a biologically targeted intervention in benign musculoskeletal disease.

## Methods and Materials

This institutional review board approved retrospective study included patients treated at a single institution who underwent revision TKA for severe arthrofibrosis with adjunctive, preoperative RT between 2023 and 2025. Exclusion criteria included periprosthetic joint infection, component malposition, or inadequate follow-up (<6 weeks).

All patients received a single fraction of external beam RT delivered within 24 hours preoperatively. The dose delivered was 8.0 Gy, prescribed to the 100% isodose line, using high energy photons and an anterior-posterior/posterior-anterior beam arrangement. RT was planned using computed tomography–based 3-dimensional conformal planning to ensure accurate volumetric coverage of the periarticular soft tissues.

**Figure 1:**
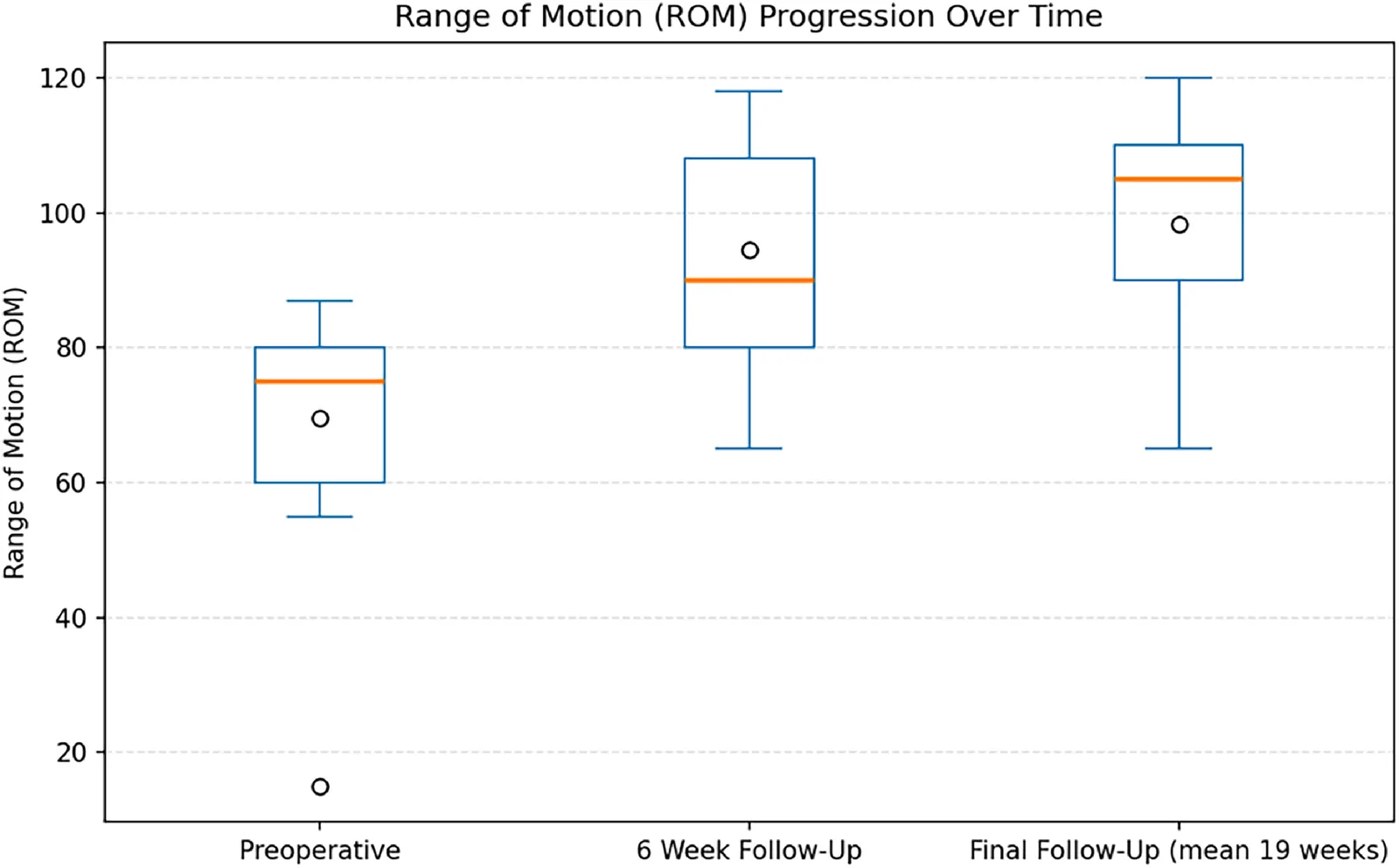
Distribution of range of motion (ROM) timepoints. Box and Whisker plots demonstrate the progression of knee ROM from the preoperative baseline through the 6 week and final follow-up. ROM means increase across successive timepoints, indicating a consistent postoperative improvement. Boxes represent the interquartile range (25th-75th percentiles). Whiskers indicate the data range excluding outliers. The horizontal line within each box denotes the median value, and the central circle denotes the mean value. The progressive increase in both measures of central tendency reflect meaningful functional gains following surgical intervention.

All surgeries were performed with a medial parapatellar approach, with extensive scar tissue resection from the medial gutter, lateral gutter, and suprapatellar regions of the knee joint (Fig. 1). Medial collateral ligament, lateral collateral ligament, and popliteus were released from their origins on the femur and resected to prevent recurrence of adhesions and to prevent mismatch of the knee kinematics with the hinged knee components. A cobb was used to strip all soft tissue attachments from the posterior femur proximally until the metadiaphyseal region of the femur was reached. Prior total knee components were then removed. Goals of reconstruction were to create a balanced gap in extension, and to downsize the femoral component to the smallest size allowed for the patient’s anatomy, thus loosening up the flexion gap and improving motion. All patients received Zimmer Rotating Hinge Knee with press-fit stems with metaphyseal cone fixation. Postoperatively, patients were kept in a knee immobilizer at all times for 2 weeks and then standardized physical therapy protocols were performed twice a week for a minimum of 6 weeks.

Clinical outcomes included passive ROM measured at preoperatively, 6 weeks, and at final follow-up. Patient-reported outcomes were assessed using the PROMIS Physical Function (PF), PROMIS Pain Interference (PI), PROMIS Global Physical Health (GPH), and Knee injury and Osteoarthritis Outcome Score for Joint Replacement (KOOS Jr) instruments at baseline and postoperatively. PROMIS is a standardized score for patient-reported health: PF scores assess a patient’s ability to perform physical activities and PI scores assess the extent to which pain interferes with physical, emotional, and social activities. GPH scores assess overall physical health, including function, health perceptions, and pain. KOOS Jr is specific to the knee, and measures pain, stiffness, and functional limitations related to knee.

Safety outcomes included postoperative complications and radiation-related adverse events. Paired *t* tests were performed to compare pre- and postoperative outcomes, with significance defined as *P* < .05.

## Results

### Patient characteristics

Thirteen patients were included in the analysis (Table 1). The majority of patients were female. The mean age was 63. Patients had undergone a mean of 2 prior surgeries (range, 1-6), and 7 had undergone prior manipulation under anesthesia. The mean follow-up duration was 19 weeks (range, 6-88).

**Table 1:** Patient demographics and clinical characteristics

Variable	Value
Patients (no.)	13
Mean Age (y)	63 (range, 52-83)
Sex	9 female, 4 male
Knee laterality	8 right, 5 left
Prior surgeries	mean 2 (range, 1-6)
Prior MUA	6 yes, 7 no
Flexion contracture ≥10°	8
Follow-up duration (weeks)	mean 19 (range, 6-88)

*Abbreviation:* MUA = Manipulation Under Anesthesia.

Demographic and clinical features including age, sex, laterality, prior surgical history, and duration of follow-up.

### ROM outcomes

**Figure 2:**
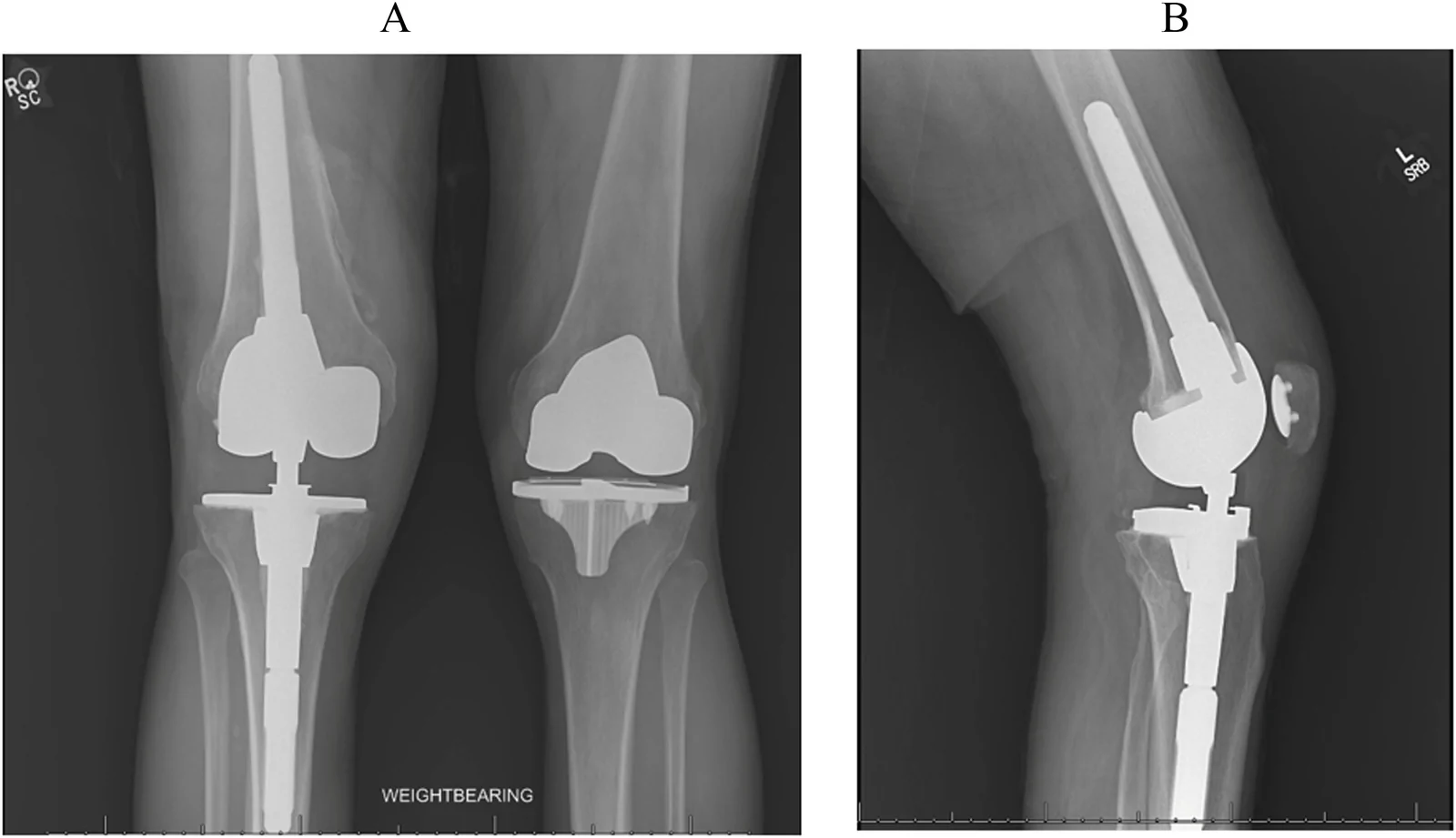
Postoperative radiographic evaluation of rotating-hinge knee arthroplasty. (A) Anteroposterior (B) Lateral weightbearing radiographs demonstrate a well-aligned Zimmer Rotating Hinge Knee prosthesis. Long press-fit stems provide diaphyseal stability, augmented by metaphyseal cone fixation to manage bone loss and guarantee rigid construct structural integrity following extensive soft tissue debridement.

**Figure 3:**
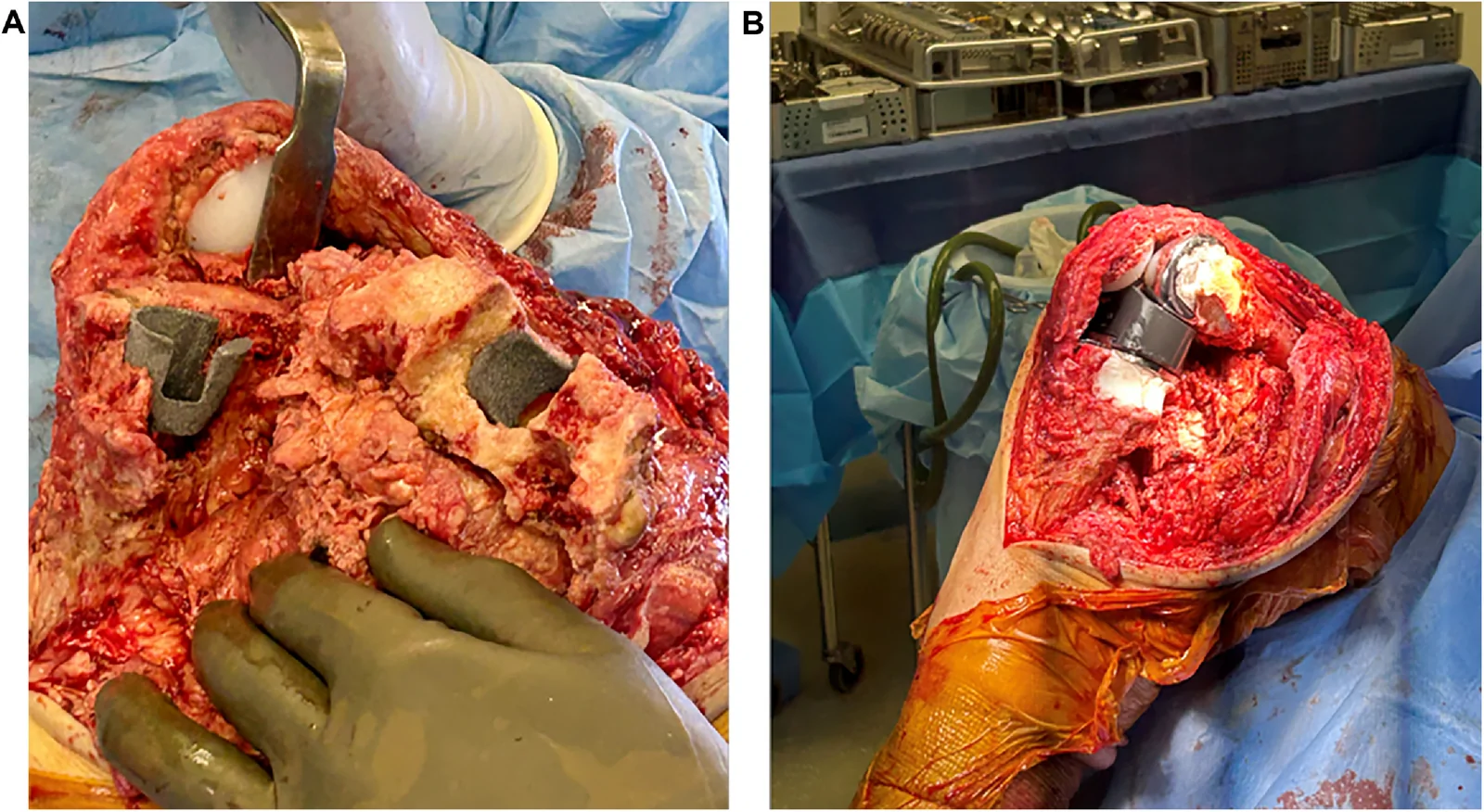
Intraoperative demonstration of radical scar tissue resection and component insertion. (A) Anterior view of the distal femur following extensive, radical resection of arthrofibrotic scar tissue from the medial and lateral gutters. All soft tissue attachments have been meticulously stripped proximally to the metadiaphyseal region to eliminate mechanical restriction and eradicate sites of fibrotic recurrence. Metaphyseal cones are seen securely seated in position. (B) Final intraoperative construct following seating of the rotating-hinge femoral and tibial components, demonstrating complete clearance of the flexion and extension pathways before closure.

The mean preoperative arc of motion was 69.5° (extension deficit 13.5°, flexion 83°). The mean ROM improved to 94.5° (extension deficit 3.4°, flexion 97.9°) at 6 weeks (*P* < .01 vs preoperative). This improved further to 98.3° (extension deficit 2.5°, flexion 100.8°) at final follow-up (*P* < .01). The mean overall gain in ROM across the cohort was 28.8° (range, 69.5° to 98.3°). ROM outcomes are summarized in Table 2 and illustrated in Figure 2, with stable postoperative mechanical alignment and construct structural integrity confirmed radiographically (Fig. 3). No radiation-related adverse effects were observed. No patients required reoperation during the follow-up period.

**Table 2:** Range of motion outcomes, passive knee range of motion (ROM) measurements obtained preoperatively, at 6 weeks postoperatively, and at final follow-up

Timepoint	Mean ROM (°)	Mean max extension (°)	Mean max flexion (°)	*P* value
Preoperative	69.5	13.5	83.0	—
6 weeks	94.5	3.4	97.9	.004
Final follow-up	98.3	2.5	100.8	.001

*Abbreviation:* ROM = range of motion.

Significance assessed against preoperative baseline.

### Patient-reported outcomes

Patient-reported outcomes were available for 12 of the 13 included patients and demonstrated significant improvements over time in most domains (Table 3).

**Table 3:** Patient-reported outcome measures, mean changes in KOOS Jr, and PROMIS domain scores from preoperative baseline to 3-month follow-up

Measure	No. pairs	Mean change at 3 months	*P* value
KOOS	10	25.3	.004*
PROMIS Physical Function	9	7.8	.013*
PROMIS Pain Interference	9	−10.1	.017*
PROMIS Global Physical Health	9	5.8	.194

*Abbreviation:* KOOS = Knee injury and Osteoarthritis Outcome Score Joint Replacement; PROMIS = Patient-Reported Outcomes Measurement Information System.

Patient-Reported Outcomes Measurement Information System (PROMIS) PF scores significantly improved from 34.2 preoperatively to 42 at 3-month follow-up (*P* = .013). PROMIS PI scores significantly decreased from 67.7 to 57.6 (*P* = .017), reflecting reduced pain interference. PROMIS GPH scores improved from 39.3 to 45.1, though this did not reach statistical significance (*P* = .194). Detailed patient-reported outcomes and domain scores over time are presented in Table 4, Table 5.

**Table 4:** Knee injury and Osteoarthritis Outcome Score for Joint Replacement (KOOS Jr) scores over time

Timepoint	No.	Mean	SD
Preoperative	12	36.4	11.6
3 months	10	61.7	12.4
12 months	2	72.0	1.9

**Table 5:** Patient-Reported Outcomes Measurement Information System (PROMIS) domain scores over time

PROMIS domain	Timepoint	No.	Mean	SD
Physical Function	Preoperative	10	34.2	4.5
	3 months	9	42.0	6.9
	12 months	3	40.9	8.2
Pain Interference	Preoperative	10	67.7	7.3
	3 months	9	57.6	8.9
	12 months	3	59.9	6.3
Global Physical Health	Preoperative	10	39.3	8.6
	3 months	9	45.1	9.9
	12 months	3	44.1	4.0

Physical Function, Pain Interference, and Global Physical Health scores collected preoperatively and at postoperative follow-up time points.

*Abbreviation:* PROMIS = Patient-Reported Outcomes Measurement Information System.

KOOS Jr scores significantly increased from 36.4 preoperatively to 61.7 at 3 months (*P* = .004). KOOS Jr score change over time is shown in Table 4.

## Discussion

This retrospective cohort study demonstrates that preoperative RT in combination with revision TKA for severe arthrofibrosis is associated with significant improvements in both objective ROM and patient-reported outcomes. Patients achieved a mean gain of 28.8° in total arc of motion, approaching restoration of near-full extension and flexion maintained at a mean follow-up of 19 weeks. Importantly, these objective improvements translated into meaningful functional and symptomatic benefits: KOOS Jr increased by 25.3 points, PROMIS PF improved by 7.8 points, and PROMIS PI decreased by 10.1 points. Collectively, these findings indicate that preoperative RT may not only improve joint motion but also enhance pain and functional recovery as experienced by patients.

Our results are consistent with prior clinical series reporting the benefit of perioperative RT for refractory arthrofibrosis. Farid et al^10^ observed a mean gain of 57° and a 28° reduction in flexion contracture following preoperative RT and constrained revision arthroplasty. Schneider et al^12^ reported long-term durability, with approximately 60° ROM gains at 6 years and 97% implant survival at 10 years in 60 patients undergoing preoperative RT and rotating-hinge revision. Smith et al^11^ conducted a comparative analysis, finding a 25° gain in flexion in RT-treated patients versus 17° in controls, though the difference did not reach statistical significance. Our results, with a mean gain of 28.8°, and fall between Smith et al’s comparative findings and the larger series of Farid et al and Schneider et al.

A key contribution of this study is the integration of validated patient-reported outcomes. While previous work has largely focused on ROM, our data demonstrate concordant improvements in KOOS Jr and PROMIS domains, confirming that gains in joint motion correspond with meaningful improvements in PF and PI. Notably, PROMIS Global Physical Health improved modestly but did not reach statistical significance, possibly because of the relatively short follow-up period and small sample size.

Mechanistically, RT is believed to target proliferating fibroblasts and myofibroblasts in the perioperative window, thereby reducing excessive scar tissue formation. Preclinical studies have shown that preoperative RT inhibits fibroblast contractility and extracellular matrix synthesis,^7^ whereas animal models of joint contracture demonstrate a surge of radiosensitive myofibroblasts in the early postoperative period.^4^ These biologic insights provide a rationale for the observed clinical improvements.

Safety outcomes were also favorable, with no radiation-related adverse events or significant postoperative complications observed. These findings align with prior studies, which similarly report no increase in wound complications, infections, or implant loosening attributable to RT.^10^, ^11^, ^12^ Given the theoretical risks of impaired healing or late fibrosis, long-term monitoring remains warranted, though the available clinical evidence supports the safety of single-fraction RT in this context.

This study has limitations. The retrospective design, small sample size, and absence of a control group limit causal inference. Additionally, follow-up was limited to a mean of 19 weeks, precluding assessment of long-term durability, and given the theoretical risks of impaired healing or late fibrosis, long-term monitoring remains warranted. However, large retrospective patient series evaluating revision arthroplasty without RT do report 12-week outcomes in addition to longer follow-up periods and demonstrate similar ROM outcomes at 12 weeks and at 1 year or beyond.^13^ Nonetheless, the consistency of ROM with prior series supports the potential utility of RT as an adjunct in managing refractory arthrofibrosis. Furthermore, the addition of favorable patient-reported outcomes using validated instruments represents a significant contribution to the literature evaluating the expanding indications for RT in benign diseases.

## Conclusions

Preoperative RT appears to be a safe and potentially effective adjunct to revision TKA in patients with severe idiopathic arthrofibrosis. In this cohort, patients achieved a mean gain of 28.8° in ROM, alongside significant improvements in KOOS Jr, PROMIS PF, and PROMIS PI. Importantly, no radiation-related complications or reoperations were observed.

These findings build on prior case series and long-term observational studies, while uniquely demonstrating parallel improvements in both objective motion and validated patient-reported outcomes. Further prospective, controlled studies are warranted to confirm efficacy, define optimal timing and dose, and evaluate long-term durability and patient-centered benefits of preoperative RT in arthrofibrosis management.

## Disclosures

Dr. Cody Green reports a relationship with DePuy Synthes that includes consulting or advisory roles. Dr. George Haidukewych reports a relationship with DePuy Orthopaedics Inc. and Smith & Nephew Inc. that includes consulting or advisory roles and reports a relationship with Zimmer Biomet and DePuy Orthopaedics Inc. that includes royalties or licenses. Dr. Justin Rineer reports a relationship with Varian Medical Systems Inc. that includes serving as a paid member of a data safety monitoring board for an ongoing clinical trial. The remaining authors (Jason Liu, Dr. Patrick Kelly, and Dr. Amish P. Shah) declare that they have no known competing financial interests or personal relationships.
